# A novel high-throughput screen for identifying lipids that stabilise membrane proteins in detergent based solution

**DOI:** 10.1371/journal.pone.0254118

**Published:** 2021-07-12

**Authors:** Cristina Cecchetti, Jannik Strauss, Claudia Stohrer, Claire Naylor, Edward Pryor, Jeanette Hobbs, Simon Tanley, Adrian Goldman, Bernadette Byrne

**Affiliations:** 1 Department of Life Sciences, Imperial College London, London, United Kingdom; 2 Astbury Centre for Structural and Molecular Biology, University of Leeds, Leeds, United Kingdom; 3 Molecular Dimensions, Sheffield, United Kingdom; 4 Anatrace, Maumee, Ohio, United States of America; 5 Molecular Dimensions, Sheffield, United Kingdom; 6 Molecular Dimensions, Sheffield, United Kingdom; 7 MIBS, Biological and Environmental Sciences, University of Helsinki, Helsinki, Finland; University of Cambridge, UNITED KINGDOM

## Abstract

Membrane proteins have a range of crucial biological functions and are the target of about 60% of all prescribed drugs. For most studies, they need to be extracted out of the lipid-bilayer, *e*.*g*. by detergent solubilisation, leading to the loss of native lipids, which may disturb important protein-lipid/bilayer interactions and thus functional and structural integrity. Relipidation of membrane proteins has proven extremely successful for studying challenging targets, but the identification of suitable lipids can be expensive and laborious. Therefore, we developed a screen to aid the high-throughput identification of beneficial lipids. The screen covers a large lipid space and was designed to be suitable for a range of stability assessment methods. Here, we demonstrate its use as a tool for identifying stabilising lipids for three membrane proteins: a bacterial pyrophosphatase (*Tm*-PPase), a fungal purine transporter (UapA) and a human GPCR (A_2A_R). A_2A_R is stabilised by cholesteryl hemisuccinate, a lipid well known to stabilise GPCRs, validating the approach. Additionally, our screen also identified a range of new lipids which stabilised our test proteins, providing a starting point for further investigation and demonstrating its value as a novel tool for membrane protein research. The pre-dispensed screen will be made commercially available to the scientific community in future and has a number of potential applications in the field.

## 1. Introduction

Integral membrane proteins are intimately associated with biological, lipid-based membranes. They are involved in a variety of cellular processes crucial for organism survival such as catalysis, signal transduction and transport of ions or small biomolecules in and out of the cell. Due to their fundamental biological functions membrane proteins are major drug targets accounting for up to 60% of approved drugs despite making up just 20–30% of the proteome [1]. G-protein coupled receptors (GPCRs) alone are targeted by approximately 35% of all approved drugs [2]. Rational drug discovery pipelines are much more efficient and economical than classical screening approaches but rely heavily on structural data [3]. However, membrane proteins account for only ~3% of the deposited structures in the PDB [4]. Structural studies of integral membrane proteins are still very challenging as the proteins must be extracted in a structurally and functionally relevant state from their natural environment, the lipid bilayer, and bottlenecks occur at every step from expression to structure determination [5].

The use of detergents is arguably the most common and successful approach for integral membrane protein extraction and solubilisation, although it has some limitations. The inherent removal of native lipids and reconstitution in detergent micelles during this process disrupts protein-lipid interactions that are important for protein stability, organisation and function [6,7]. The physicochemical properties of detergent micelles differ substantially from lipid bilayers, making them a poor membrane mimic [8]. For example, both curvature [9] and lateral pressure [10] provided by the membrane environment are important for protein structure and function. Similarly, lateral tension also affects membrane protein function [11]. Selective protein-lipid interactions have also been extensively documented as playing crucial roles. Phospholipids binding at the dimer interface are crucial for maintenance of the functional, quaternary state of the eukaryotic purine transporter, UapA [12]. Cardiolipin (CL) has been shown to be crucial for dimerisation of the bacterial leucine transporter LeuT [13]; cholesterol (CHL) is well known to affect the function and stability of GPCRs [14]; and there is emerging evidence for the essential roles that phospholipids play in coupling of receptors to effector molecules [15].

To compensate for decreased protein stability upon solubilisation into detergent, one can screen different detergents and/or additives, or engineer more robust protein variants [16–19]. Improved stability conferred by amino acid substitutions often comes at the cost of reduced activity as protein motion is impeded by rigidifying flexible regions or locking the protein in a particular conformational state [16–19]. Although this may benefit structural characterisation, it hampers functional studies and may call into question the physiological relevance of study outcomes. Alternatively, detergent-free solubilisation strategies such as amphipols [20], bicelles [21], liposomes [22], nanodiscs [23], peptidiscs [24] and styrene-maleic acid copolymer lipid particles (SMALPs) [25] can be tried. All of these aim to better mimic the native lipid bilayer composition and/or its physicochemical properties. Nevertheless, most solubilisation platforms still require an intermediate detergent extraction step, resulting in the loss of most if not all native lipids [20–24]. Only SMALPs extract membrane proteins directly out of the lipid bilayer while preserving the surrounding native lipid environment, but their use in downstream experiments is limited due to the formation of heterologous complexes that exhibit sensitivity to low pH (< 6.5) and divalent cations (> 5 mM) [25,26]. A simple and potentially effective way to restore structural integrity and protein function is the relipidation of integral membrane proteins after detergent extraction. Such an approach was essential for the structural characterisation of a broad range of targets such as electron transport complexes [27], ion pumps [28], ion channels [29], transporters [30] and GPCRs [31]. Unfortunately, screening is expensive and laborious, since it is difficult to know which lipids are critical for an individual membrane protein.

Testing the effect of lipids on protein stability, function or crystal growth only requires a few hundred micrograms, which is much less than the prevailing commercially available aliquot sizes. We have therefore designed a multi-purpose microplate screen containing sufficient amounts of 31 different lipids or lipid mixtures to help the scientific community sample a broad range of lipids at an affordable cost and with minimal effort. It is, as far as aware, the first such screen. Here, we report the application of this lipid screen to identify stabilising lipids of three different integral-membrane proteins by differential scanning fluorimetry (DSF).

Our test proteins are a membrane-bound pyrophosphatase (M-PPase) from the thermophilic bacterium *Thermotoga maritima* (*Tm*-PPase), a eukaryotic purine transporter from *Aspergillus nidulans* (UapA) and a member of the GPCR family, the human A_2A_ receptor (A_2A_R). We chose these proteins as they were under investigation in our laboratories; they can be expressed in suitable amounts and, most importantly, they are a test set of challenging membrane proteins. They have different folds (from 7 to 16 transmembrane helices), different topologies and different modes of action [32–35]. M-PPases utilise the pyrophosphate pool to generate electrochemical gradients across membranes in plants, prokaryotes and protist parasites, thereby facilitating their survival during low-energy periods, high-stress conditions and varying osmotic environments [36]. Their potential as drug targets to fight severe parasitic diseases such as malaria, the African sleeping sickness, or toxoplasmosis is currently being investigated [32]. Additionally, M-PPase overexpression in transgenic plants leads to improved drought resistance and is a potential point of action to address worsening trends of global-warming-induced crop loss, which poses a threat to the food supply of millions of people [37,38]. UapA is a high affinity, high capacity symporter, responsible for the uptake of uric acid-xanthine/H^+^ in *Aspergillus nidulans* [39]. It is the most extensively characterised member of the Nucleobase-Ascorbate Transporter (NAT) family, which includes proteins that transport essential metabolites such as nucleobases in bacteria, plants and fungi and ascorbate (vitamin C) in mammals [40]. UapA represents not only an important model protein to study the members of the NAT family, but also a promising drug target for *Aspergillosis*, a fungal lung infection with high mortality rate in immunocompromised patients [33]. A_2A_R is one of four human adenosine receptor subtypes (A_1_, A_2A_, A_2B_, A_3_), all of which belong to the family of GPCRs [41]. It is expressed in numerous human tissues where it is involved in the regulation of myocardial blood flow, has regulatory functions in the adaptive immune system and plays a role in the regulation of dopamine and glutamate responses in the brain [41]. Because of its involvement in various physiological and pathological processes, A_2A_R represents a very promising drug target, for example being investigated in inflammation-related disease such as asthma [34], and neurodegenerative disease such as Parkinson’s disease [35].

## 2. Results

### Rationale of the lipid screen

The lipidic environment of the biological membrane not only acts as the scaffold in which membrane proteins are embedded but can also affect protein stability and correct folding as well as oligomerisation and function. Often, choosing the best lipids and lipid concentration to stabilise a specific membrane protein for functional or structural analysis relies on a trial and error process and it can be challenging, expensive and time consuming. The lipid screen aims to simplify this process and allow cost effective testing of a large lipid space. It was designed to be a versatile tool for lipid screening and can be used for stability testing, functional assays and structural characterisation for example *via* protein crystallisation using vapour diffusion or high lipid-detergent (HiLiDe) methods [42].

The screen contains 23 unique lipids of synthetic and natural origin (Table 1), including a range of different phospholipids which have been shown to co-purify and co-crystallise with and have functional effects on a range of membrane proteins proteins (see references cited in Table 1 for more details). We incorporated several lipid classes including phosphatidylethanolamines (PEs), phosphatidylcholines (PCs), phosphoglycerides (PGs), phosphatidylserines (PSs), phosphatidic acids (PAs), cardiolipins (CLs) and sphingolipids (SLs).

**Table 1:** Lipid screen design containing 31 different lipids or lipid mixtures.

Well	Lipid	Abbreviation	Amount	Comment	References
A1	1,2-Dioleoyl-*sn*-Glycero-3-Phosphoethanolamine	DOPE	0.3 mg	Phosphatidylethanolamine (PE)	[43]
A2					
A3					
A4	1-Palmitoyl-2-Oleoyl-*sn*-Glycero-3-Phosphoethanolamine	POPE	0.3 mg	Phosphatidylethanolamine (PE)	[12]
A5					
A6					
A7	1,2-Dipalmitoyl-*sn*-Glycero-3-Phosphoethanolamine	DPPE	0.3 mg	Phosphatidylethanolamine (PE)	[43]
A8					
A9					
A10	1-Palmitoyl-2-Oleoyl-*sn*-Glycero-3-Phosphocholine	POPC	0.3 mg	Phosphatidylcholine (PC)	[44]
A11					
A12					
B1	1,2-Dioleoyl-*sn*-Glycero-3-Phosphocholine	DOPC	0.3 mg	Phosphatidylcholine (PC)	[45]
B2					
B3					
B4	1,2-Dimyristoyl-*sn*-Glycero-3-Phosphocholine	DMPC	0.3 mg	Phosphatidylcholine (PC)	[46]
B5					
B6					
B7	1,2-Dihexadecanoyl-*sn*-Glycero-3-Phosphocholine	DPPC	0.3 mg	Phosphatidylcholine (PC)	[47]
B8					
B9					
B10	1-Myristoyl-2-Hydroxy-*sn*-Glycero-3-Phosphocholine	LMPC	0.3 mg	Phosphatidylcholine (PC)	[48]
B11					
B12					
C1	1-Palmitoyl-2-Oleoyl-*sn*-Glycero-3-Phosphoglycerol (sodium Salt)	POPG-Na	0.3 mg	Phosphoglyceride (PG)	[49]
C2					
C3					
C4	1,2-Dipalmitoyl-*sn*-Glycero-3-Phospho-1’-*rac*-Glycerol (sodium salt)	DPPG-Na	0.3 mg	Phosphoglyceride (PG)	[49]
C5					
C6					
C7	1-Palmitoyl-2-Oleoyl-*sn*-Glycero-3-Phospho-L-Serine (sodium salt)	POPS	0.3 mg	Phosphatidylserine (PS)	[50]
C8					
C9					
C10	1,2-Dioleoyl-*sn*-Glycero-3-Phospho-L-Serine (sodium salt)	DOPS-Na	0.3 mg	Phosphatidylserine (PS)	-
C11					
C12					
D1	1,2-Didecanoyl-*sn*-Glycero-3-Phosphate (sodium salt)	10:PA-Na	0.3 mg	Phosphatic acid (PA)	[51]
D2					
D3					
D4	1-Palmitoyl-2-Oleoyl-*sn*-Glycero-3-Phosphate (sodium salt)	16:0–18:1 PA (POPA)	0.3 mg	Phosphatic acid (PA)	-
D5					
D6					
D7	*E*. *coli* Polar Lipid Extract	EPL	0.3 mg	Mixture of PE (67%), PG (23.2%) and CA (9.8%)	[52]
D8					
D9					
D10	Cholesteryl Hemisuccinate Tris Salt	CHS	0.3 mg	Sterol	[53]
D11					
D12					
E1	Cholesterol	CHL	0.3 mg	Sterol	[54]
E2					
E3					
E4	1’,3’-bis[1,2-dimyristoyl-*sn*-glycero-3-phospho]-glycerol (sodium salt)	14:0 CL	0.3 mg	Cardiolipin (CL)	[13]
E5					
E6					
E7	1’,3’-bis[1,2-dioleoyl-*sn*-glycero-3-phospho]-glycerol (sodium salt)	18:1 CL	0.3 mg	Cardiolipin (CL)	[13]
E8					
E9					
E10	Porcine Polar Brain Lipid Extract	PBL	0.3 mg	Mixture of PC (12.6%), PE (33.1%), PI (4.1%), PS (18.5%), PA (0.8%) and unknown (30.9%)	[55]
E11					
E12					
F1	Egg Sphingomyelin (chicken)	SM	0.3 mg	Mixture of sphingomyelins (SM) with 16:0 SM (86%), 18:0 SM (6%), 22:0 SM (3%), 24:1 SM (3%), unknown (2%)	[56]
F2					
F3					
F4	Monomyristolein	7.7MAG	0.3 mg	Monoacylglycerol	[57]
F5					
F6					
F7	Monoolein	9.9MAG	0.3 mg	Monoacylglycerol	[58]
F8					
F9					
F10	Lipid mix I	POPC:POPG: POPE (3:1:1)*	0.3 mg	Used for structure solution of several ion channels	[59–61]
F11					
F12					
G1	Lipid mix II	POPC:POPS (4:1)*	0.3 mg	Simple mimic of the membrane inner lipid leaflet	-
G2					
G3					
G4	Lipid mix III	POPG:POPE (3:1)*	0.3 mg	Simple mimic of Gram-positive bacterial membranes	-
G5					
G6					
G7	Lipid mix IV	POPG:POPE (1:3)*	0.3 mg	Simple mimic of Gram-negative bacterial membranes	-
G8					
G9					
G10	Lipid mix V	DMPC:CHL (2:1)*	0.3 mg	Simple mimic of eukaryotic plasma membranes	-
G11					
G12					
H1	Lipid mix VI	POPC:CHL (5:1)*	0.3 mg	Simple mimic of nerve cell membranes	-
H2					
H3					
H4	Lipid mix VII	CHL:POPC:SM (1.8:1:1)*	0.3 mg	Simple mimic of erythrocyte membranes	-
H5					
H6					
H7	Lipid mix VIII	POPC:POPE (1:1)*	0.3 mg	Simple mimic of mitochondrial membranes	-
H8					
H9					
H10	Blank	Blank	0 mg	Control	-
H11					
H12					

* molar ratios.

CHL is known to associate with a range of mammalian membrane proteins in particular GPCRs [14] and transporters [54,62,63]. The native cholesterol molecule is relatively insoluble in aqueous solution, so researchers tend to use the more soluble salt, cholesterol hemisuccinate (CHS) as an additive to protein solutions. We included both forms in our screen. CL is known to form associations with a range of membrane proteins particularly respiratory complexes [64] but also transporters [13]. Monoacylglycerols (MAGs) are widely used for lipidic cubic phase (LCP) crystallisation [58,57]; we included a couple of examples of these molecules in our initial screen as a means of assessing whether we were able to identify the best MAG for stabilisation and thus LCP crystallisation of our test proteins. We also included various lipid extracts from natural sources as well as 8 unique defined lipid mixtures allowing a controlled, yet more complex lipid environment more similar to eukaryotic and prokaryotic membranes. The lipids were selected based on an extensive literature scan using the LipidMAPS database (https://www.lipidmaps.org), which lists specific lipids bound to membrane proteins identified in native mass spectrometry and lipidomics analysis, and the PDB database (https://www.rcsb.org) to pinpoint the most frequent lipids found in protein structures. Additionally, material and methods sections of selected structural biology research articles were studied to identify the lipids and lipid mixtures commonly used in purification steps and crystallisation trials. We also considered lipid cost and stability in our selection, for example no phosphatidylinositols (PIs) have been included in order to keep the overall cost of the screen down.

### Use of the lipid screen

The solubilisation of lipids in 3% detergent was incomplete using the protocol described above as some wells showed remaining precipitate. Lipid solubilisation can be improved by increasing the detergent concentration if this does not interfere with downstream processes. Alternatively, more extensive solubilisation protocols with higher temperatures, addition of magnetic stirring bars, longer incubation periods, or sonication can be used. However, for the identification of stabilising lipids the exact final lipid concentration is irrelevant as long as sufficient amounts of lipids are solubilised and, indeed, by Le Chateliers’ principle some of the precipitated lipid may redissolve if the lipid is bound to the membrane protein introduced. The lipid amount dispensed in the screen was therefore chosen to be suitable for a range of applications for example HiLiDe crystallisation trials to provide a cost effective way of screening many different lipids (see Conclusion). Only one screen was used to assess the effect of lipids on protein stability for all test proteins in triplicates following our protocol, further highlighting the cost effectiveness of our screen. In the next paragraph we report on the effects of the tested lipids on protein stability, demonstrating sufficient solubilisation following our protocol for this test purpose.

### Effects of lipids on protein stability

**Fig 1:**
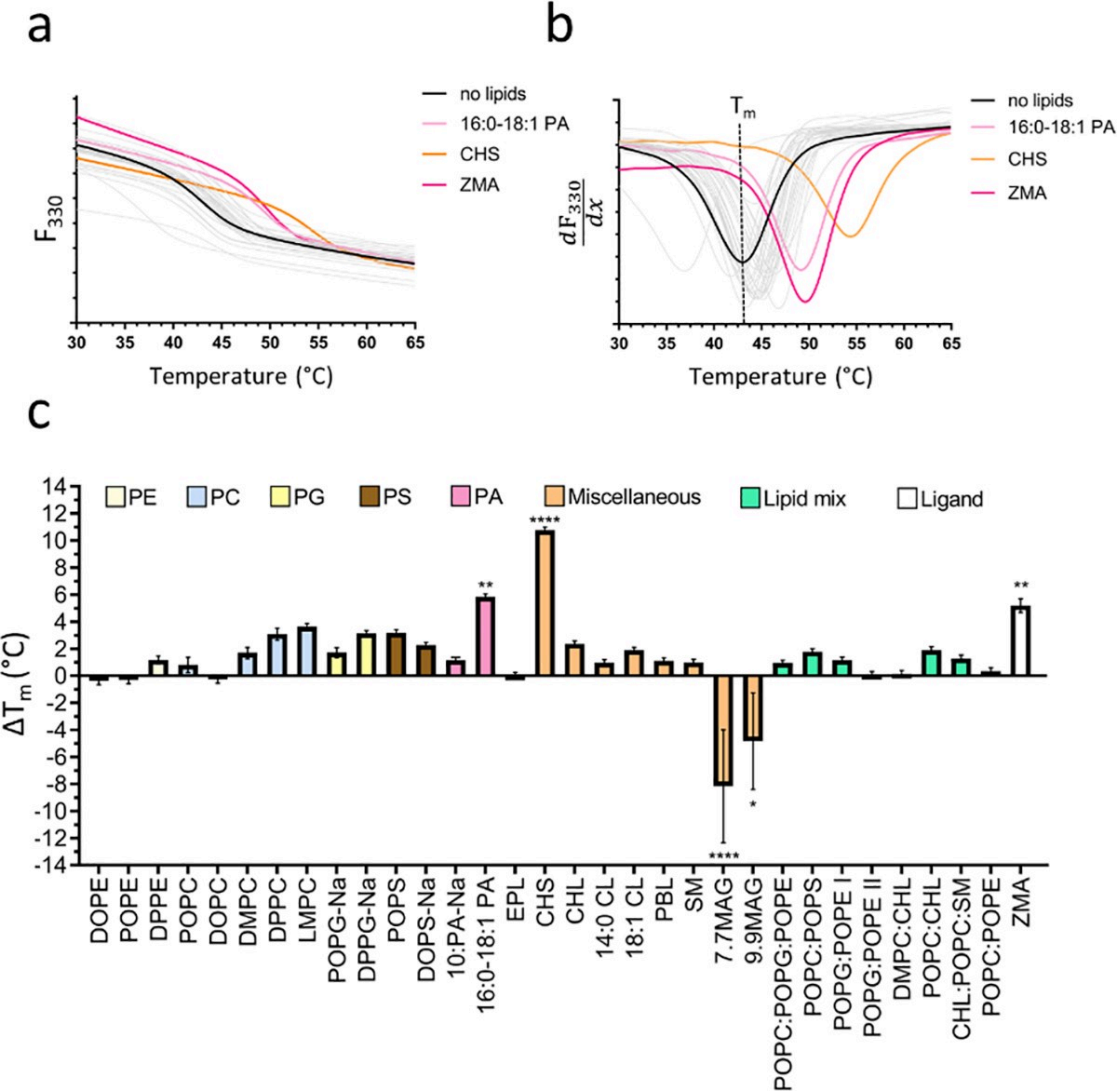
Lipid screening of A_2A_R by nanoDSF. (**a**) F_330_ signal of the no lipids reference sample (black line) and a representative set of stabilising lipids (coloured lines) during the melting scan. Grey lines show the fluorescence traces of the other tested lipids. (**b**) First derivative of the F_330_ signal highlighting the fluorescence change indicative of protein unfolding. The T_m_ can be determined at the curve peak, here shown for the no lipids reference sample as dotted line. (**c**) ΔT_m_ of detergent-solubilised to relipidated sample shown for all tested lipids, which are sorted and colour coded according to their lipid class. Panel a-b show the traces of a representative nanoDSF scan. Panel c shows the ΔT_m_ and corresponding standard error of the mean (SEM) based on three technical repeats. The asterixis represent the p-value (* = 0.0332, ** = 0.021, *** = 0.002, **** = 0.0001) obtained by a one-way ANOVA followed by a Dunnet test to correct for multiple comparison in GraphPadPrism 7.

**Fig 2:**
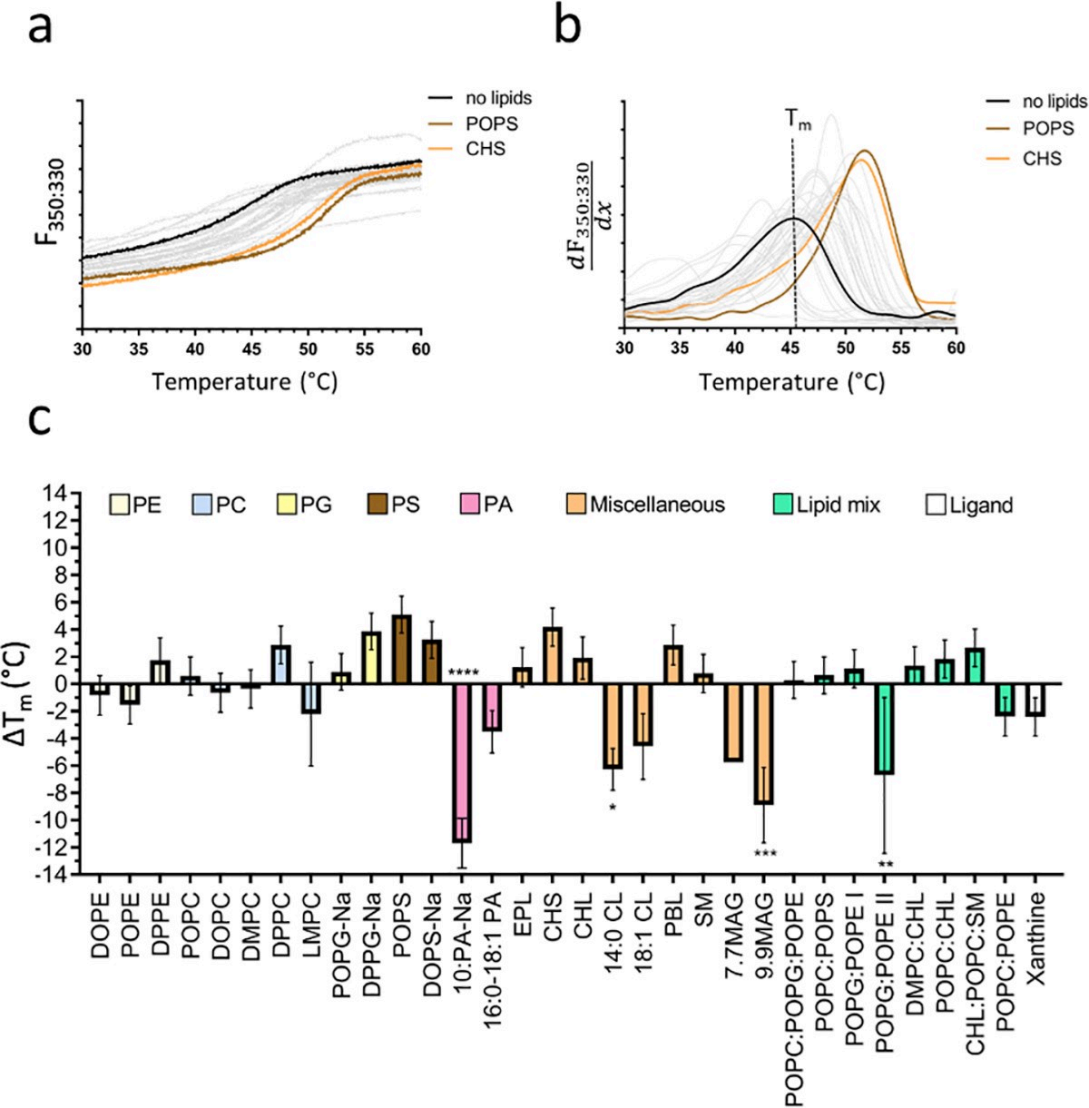
Lipid screening of UapA by nanoDSF. (**a**) F_350:330_ signal of the no lipids reference sample (black line) and a representative set of stabilising lipids (coloured lines) during the melting scan. Grey lines show the fluorescence traces of the other tested lipids. (**b**) First derivative of the F_350:330_ signal signal highlighting the fluorescence change indicative of protein unfolding. The T_m_ can be determined at the curve peak, here shown for the no lipids reference sample as dotted line. (**c**) ΔT_m_ of detergent-solubilised to relipidated sample shown for all tested lipids, which are sorted and colour coded according to their lipid class. Panel a-b show the traces of a representative nanoDSF scan. Panel c shows the ΔT_m_ and corresponding SEM based on three technical repeats. The asterixis represent the p-value (* = 0.0332, ** = 0.021, *** = 0.002, **** = 0.0001) obtained by a one-way ANOVA followed by a Dunnet test to correct for multiple comparison in GraphPadPrism 7.

**Fig 3:**
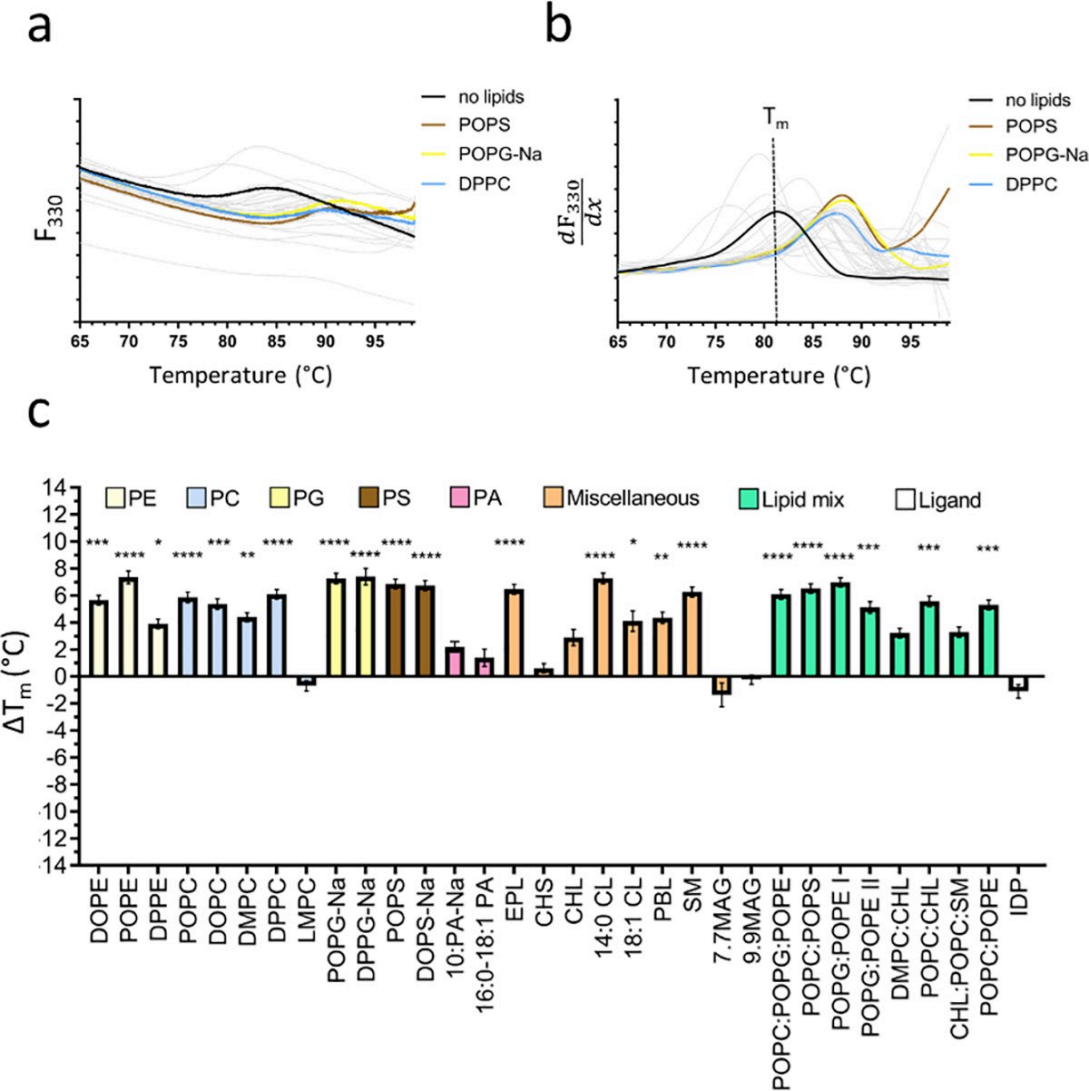
Lipid screening of *Tm*-PPase by nanoDSF. (**a**) F_330_ signal of the no lipids reference sample (black line) and a representative set of stabilising lipids (coloured lines) during the melting scan. Grey lines show the fluorescence traces of the other tested lipids. (**b**) First derivative of the F_330_ signal highlighting the fluorescence change indicative of protein unfolding. The T_m_ can be determined at the curve peak, here shown for the no lipids reference sample as dotted line. (**c**) ΔT_m_ of detergent-solubilised to relipidated sample shown for all tested lipids, which are sorted and colour coded according to their lipid class. Panel a-b show the traces of a representative nanoDSF scan. Panel c shows the ΔT_m_ and corresponding SEM based on three technical repeats. The asterixis represent the p-value (* = 0.0332, ** = 0.021, *** = 0.002, **** = 0.0001) obtained by a one-way ANOVA followed by a Dunnet test to correct for multiple comparison in GraphPadPrism 7.

The thermal denaturation of our test proteins was recorded by the change of the intrinsic fluorescence at 330 nm and 350 nm caused by local changes of the tryptophan (and tyrosine) environment during unfolding. The unfolding behaviour of relipidated protein was compared to a reference sample solubilised in detergent (no lipids) by the means of the difference in the apparent melting temperature (ΔT_m_) to assess the effect of different lipids in the lipid screen on protein stability (Figs 1–3). The F_330_ and F_350_ signal recorded during the melting scans showed a clear transition (unfolding) for A_2A_R at 43.3 ± 0.2°C (Fig 1A and 1B) and for *Tm*-PPase at 81.1 ± 0.3°C (Fig 3A and 3B). The monitoring at two wavelength (330/350 nm) allows the detection of emission peak shifts in addition to fluorescence intensity changes upon protein unfolding and fluorescence background becomes negligible when analysing the F_350:F330_ ratio. Therefore, even small differences in the local tryptophan/tyrosine environment that are not detectable in single wavelength measurements can be resolved using data obtained at 330 nm and 350 nm simultaneously. For UapA, the F_350:F330_ ratio was more informative than F_330_ or F_350_ signal alone and showed a clear transition at 46.3 ± 1.1°C (Fig 2A and 2B).

MAG’s were the only lipid class consistently showing destabilising (or no) effects on protein stability across all test proteins despite their success in the structure determination of membrane proteins using LCP (Figs 1–3C). These lipids were specifically designed to form a highly curved mesophase at a certain lipid to aqueous phase ratio [57,58]. The low concentration of the MAGs used here is clearly not suitable for identification of suitable LCP matrices and will be replaced in future versions of the screen with other molecules such as 10:0 PC and 18:0 PC, which can be used to test the effect of different alkyl chain lengths and membrane curvature on membrane proteins [65], to further widen the lipid space tested.

#### A_2A_R

The protein-lipid interplay has been extensively studied for GPCRs with CHL [14]. CHL binding is reported to lead to conformational stabilisation of several GPCRs [66–68]. Addition of the more water soluble analogue CHS was previously shown to improve A_2A_R stability and was crucial for purification and initial structure determination, which led to the identification of three different cholesterol binding sites [53,69]. Due to the well documented effects of CHL and CHS on GPCR stability, A_2A_R serves as a control protein to test the ability of the lipid screen to identify stabilising lipids.

Ligand binding and high concentrations of NaCl were shown to further stabilise A_2A_R [53]. Here, A_2A_R was purified in the absence of CHS to test its effect on protein stability following our protocol for high-throughput lipid screening using the lipid screen. As expected, CHS stabilised the receptor with a ΔT_m_ of +10.8 ± 0.2°C (Fig 1C), in good agreement with previously determined values [69]. The next best hit was 16:0–18:1 PA with a ΔT_m_ of +5.8 ± 0.2°C (Fig 1C). So far, no functional or thermostabilising effects have been reported for PA’s on A_2A_R. Besides CHL, several ordered lipid chains most likely originating from the LCP were found in A_2A_R crystal structures, of which some seem to form specific interactions [69]. Previously, phospholipids have also been shown to occupy proposed cholesterol binding pockets [53]. More recent studies further imply a functional role of anionic phospholipids such as PIP_2_ in receptor activation [15]. Phospholipids, particularly PA with a very small headgroup, might be able to bind to these lipid binding pockets and thereby stabilise the receptor in one particular conformation.

The addition of 50 μM of the A_2A_R antagonist, ZM241385 (ZMA) served as an assay control, conferring an increase in T_m_ of +5.2 ± 0.5°C as expected (Fig 1C). This value is slightly lower than previously reported [53], which is probably due to the different assay buffer composition used (no CHS but high salt and theophylline present).

#### UapA

Using the lipid screen, we identified previously unknown stabilising lipids for UapA. However, their effect was less pronounced compared to the CHS and 16:0–18:1 stabilisation of A_2A_R. All PSs tested stabilised UapA, with POPS having the biggest effect (ΔT_m_: +5.1 ± 1.4°C) (Fig 2C). Previous studies have indicated that UapA purifies in DDM in the presence of PI, PE and PC, with both PI and PE having roles in maintaining the functional dimeric state of the protein [12]. Whilst PI is too expensive to include in the screen and was thus not assessed here, PE had no effect on UapA. MD simulations showed that the PE binds less tightly than PI and the effects of both PI and PE could only be seen following delipidation and subsequent addition of the lipids [12]. Therefore, it is possible that PI and PE lipids that remain bound to the protein during isolation form tight interactions with the protein and additional PE has no significant effect. Interestingly, we did see a stabilising effect for PS. This is not a lipid identified from previous lipidomics analysis of purified UapA, suggesting it binds relatively weakly compared to PI and PE. PS does co-purify with the structurally related boron transporter from *Saccharomyces cerevisiae* (ScBOR1p) and has a role in dimer formation [50], strongly indicating that the precise effects of this lipid on UapA are worth further analysis. CHS also stabilises the protein with a ΔT_m_ of +4.2 ± 1.4°C (Fig 2C). UapA is a fungal protein and as such physiologically exposed to ergosterol, rather than the mammalian equivalent, CHL [70]. However, due to their structural similarity CHS likely substitutes for ergosterol in stabilising UapA [71]. Further analyses exploring the effects of CHL/ergosterol are warranted in the case of UapA. CL, a lipid that UapA does not encounter in the cell, markedly destabilises UapA (Fig 2C). PA’s also destabilise UapA although natively present in *Aspergillus nidulans* membranes [70]. Intriguingly, a combination of PE and PG also has a destabilising effect on the protein although DPPG alone stabilised the transporter with a ΔT_m_ of +3.9 ± 1.3°C.

The addition of the substrate, xanthine, did not stabilise UapA (Fig 2C). It is important to note that the construct we are using is a thermostabilised form of the protein (UapA-G411V_Δ1–11_) trapped in the inward facing conformation [72]. Previous analysis indicated that the presence of xanthine stabilises this construct but only in protein that had been solubilised in the comparatively harsh detergent, nonyl-b-D-glucoside (NG). In the larger micelles of DDM, used to purify the protein for analysis here, the addition of xanthine appears to have minimal effects.

#### *Tm*-PPase

*Tm*-PPase is a thermophilic protein and, as such, unfolds at much higher temperatures compared to the mesophilic UapA and A_2A_R. To date, little is known about the effects of lipids on M-PPases. Hydrolytic activity measurements show increased activity of thermophilic M-PPases after relipidation with soybean lecithin [73]. However, soybean lecithin is not part of the lipid screen due to its short shelf life. Surprisingly, the analysis of the melting scan data reveals stabilising effects of lipids on *Tm*-PPase across almost all lipid classes represented in the screen, including anionic lipids POPG and POPS, with an overall average ΔT_m_ of +4.6 ± 0.4°C (Fig 3). Such broad stabilisation across different lipid classes is most likely conferred by non-specific interactions of protein with the lipidated detergent micelle rather than specific protein-lipid interactions (see Introduction). Indeed, *Tm*-PPase was purified in a rather harsh detergent, OGNG, used routinely for structural studies, due to the intrinsic stability of the protein [74]. The lipidation of detergent micelles likely reduces the micelle curvature, leading to increased protein stability [75]. The different effect on *Tm*-PPase stability observed within anionic lipids (*e*.*g*. 16:0–18:10PA *vs*. POPS) and PC’s (*e*.*g*. LMPC *vs*. DMPC) highlights the contribution of both lipid parts, the hydrophilic head group (changed in the former pair) and the hydrophobic lipid tail (changed in the latter pair) to their overall properties and effect on proteins (Fig 3C). Beside PA’s and LMPC, only MAG’s and CHS did not stabilise *Tm*-PPase solubilised in OGNG. Conversely, CHL addition slightly improved the thermostability (ΔT_m_: +2.9 ± 0.6°C) (Fig 3C). Biophysical characterisation and atomistic simulations have previously shown that CHS is not an ideal mimic of CHL; even lesser so in its deprotonated form (pK_a,CHS_: ~5.8), which is predominantly present in the *Tm*-PPase buffer (pH: 6.5), which may explain the differences observed between the two [76].

We also tested the effect of the non-hydrolysable inhibitor IDP, which is regularly used for the functional characterisation of M-PPases and in structural studies to lock the protein in a conformational state. IDP binding to *Tm*-PPase did not result in thermostabilisation suggesting that the IDP-bound structure is not energetically more favourable than the apo-structure (Fig 3C), even though IDP is known to protect *Tm*-PPase against protein degradation [77]. Biophysical data characterising the apo-structure of *Tm*-PPase and studies of *Tm*-PPase using a combination of lipids with IDP would yield insights into why IDP did not appear to be stabilising.

## 3. Discussion and conclusion

It is becoming increasingly well established that membrane lipids play key roles in structure and function of membrane proteins. However, we are still highly dependent on detergent based extraction and isolation methods, which result in loss of lipids, for protein preparation. Here we describe the first pre-prepared, easy-to-use screen for the high-throughput identification of stabilising lipids. The lipid screen was designed as versatile tool and could also be used in association with other screening methods, for example HiLiDe [42] by varying the detergent concentration used in combination with the different lipids. Rationally, longer-chain detergents, which are in general more stabilising (DDM *versus* DM, for instance) would be expected to have additive effects on protein stability with the lipids identified through this lipid screen. Other applications adding further value to it include the use as a lipid additive source for crystallisation trials, functional analysis (*e*.*g*. in liposomes) or reconstitution studies into nanodiscs for cryo-EM experiments.

The choice of lipids to include in the screen was based on those shown to co-purify with, crystallise in complex with, or stabilise membrane proteins and also took into account the cost and stability of individual lipids. We tested this screen with three different membrane proteins and assessed their relative stabilities in the presence of the lipids by nanoDSF (Figs 1–3). Other methods could be used for screening the stability of a given protein including dye based thermal denaturation analysis with, for example, the commonly used N-[4-(7-diethylamino-4-methyl-3-coumarinyl)phenyl] maleimide (CPM) fluorochrome [78].

Our analysis confirmed the known stabilising effects of both ZM241385 and CHS on the A_2A_R providing confidence that this approach is a suitable method for identifying stabilising lipids (Fig 1C). It is important to note that the A_2A_R protein that we worked with is thermostabilised through the incorporation of specific point mutations. However, CHS is also known to be crucial for stability of the A_2A_R modified only through the truncation of the C-terminal tail [79], thus we would expect to see the same results with respect to CHS if we had used this non-mutagenized receptor form. In addition, we identified novel molecules likely to form specific interactions with and stabilising both A_2A_R (PCs) and UapA (CHS and PSs) that provide the basis for further study (Figs 1 and 2C). Intriguingly, *Tm*-PPase was stabilised by virtually every lipid within the screen (Fig 3C), a feature we suggest is caused by the non-specific partitioning of lipids into the detergent micelle, altering the micelle structure and stabilising the associated protein.

In summary, we present a novel screen suitable for the identification of lipids that stabilise individual membrane proteins. Although the basic methodology we use here is common in membrane protein research, we report on a new high-throughput system that is very straightforward, cost effective and broadly applicable. We believe our pre-dispensed screen and the described protocol for its application will be useful to the scientific community working on challenging membrane protein targets. We are working on future commercialisation in cooperation with Molecular Dimensions in order to facilitate the structural and functional analysis of stable, physiologically relevant protein samples.

## 4. Methods

### Lipid screen preparation

All lipids were purchased from Anatrace or Avanti and solubilised in chloroform or a chloroform methanol mixture if supplied as powder. POPG (1-palmitoyl-2-oleoyl-*sn*-glycero-3-phospho-1’- *rac*-glycerol) was dissolved in a 5:1 mixture of chloroform and methanol at 10 mg/mL. DPPE (1,2-Dipalmitoyl-*sn*-glycero-3-phosphoethanolamine) was dissolved in a 9:1 mixture of chloroform and methanol at 5 mg/mL. All other lipids were dissolved in chloroform to a stock concentration of 10 mg/mL. A volume equivalent to 0.3 mg of lipid or lipid mixture (Table 1) was transferred to a round-bottom glass-coated 96-well microplate (WebSeal Plate+, Thermo Scientific) in triplicates. Well H9-H12 were left empty to serve as a blank control. The solvent was evaporated overnight under a constant stream of nitrogen gas. The plates were sealed with aluminium foil under a nitrogen atmosphere and stored at -20°C in the dark until used.

### Protein expression and purification

#### A_2A_R

We expressed and purified a stabilised human A_2A_R construct (Δ317–412, 209–218 replaced with a thermostabilized apocytochrome b562 from *Escherichia coli* (BRIL)), which was successfully used for structural studies in the past [53,69]. Expression in a baculovirus expression system followed the manufacturer’s instructions for the Bac-to-Bac system (Invitrogen). *Spodoptera frugiperda* (Sf9) insect cells were infected at a cell density of 2 million cells/mL with P2 virus at a 1:2000 dilution. Infected cells were harvested after incubation for 60 hours at 27°C while shaking.

The purification is described in detail elsewhere [69]. In brief, Sf9 membranes were disrupted and harvested by repeated Dounce homogenisation and ultracentrifugation in hypotonic [10 mM MgCl_2_, 20 mM KCl and 50 mM HEPES pH 7.5] and hypertonic [0.8 M NaCl, 50 mM HEPES pH 7.5] buffers supplemented with and 1x proteoloc protease inhibitor cocktail (Expedeon). The final membranes were resuspended in hypotonic buffer supplemented with 40% (v/v) glycerol and flash frozen in liquid nitrogen for storage at -80°C until used. Prior to solubilisation, the membranes were incubated with 2 mg/mL iodoacetamide, 4mM theophylline and 1x proteoloc protease inhibitor cocktail for 30 minutes at 4°C. The receptor was then extracted using 1% (w/v) n-dodecyl-β-D-maltopyranoside (DDM) for 4 hours at 4°C, followed by centrifugation at 150,000 xg for 1 hour at 4°C. The supernatant was incubated overnight with 1.5 mL packed HisPur cobalt resin (Fisher Scientific) per litre of culture in a final buffer containing 50 mM HEPES pH7.5, 0.8 M NaCl, 10% (v/v) glycerol 1 mM theophylline and 20 mM imidazole. The resin was washed with 10 column volumes (CV) wash buffer A [50 mM HEPES (pH 7.5), 0.8M NaCl, 10 mM MgCl_2_, 10% (v/v) Glycerol, 25 mM imidazole, 0.1% (w/v) DDM, 8 mM ATP,1 mM Theophylline] followed by 4 CV wash buffer B [50 mM HEPES (pH 7.5), 0.8 M NaCl, 10% (v/v) glycerol, 50 mM imidazole, 0.05% (w/v) DDM, 1 mM theophylline]. The receptor was eluted in 9x 1 CV fractions elution buffer [25 mM HEPES pH7.5, 0.8 M NaCl, 10% (v/v) glycerol, 220 mM imidazole, 0.025 (w/v) DDM, 0.5 mM theophylline]. Elution fractions containing purified A_2A_R were combined after SDS-PAGE analysis (S1 Fig). The purified protein sample was concentrated to 2 mg/mL with a 100 kDa molecular weight cut off (MWCO) Vivaspin centrifugal concentrator (GE Healthcare), and buffer exchanged to the final assay buffer [25 mM HEPES (pH 7.5), 0.8M NaCl, 1% (v/v) glycerol, 0.01% (w/v) DDM, 0.5 mM theophylline] using a PD10 desalting column (GE Healthcare). The final protein concentration was 1 mg/mL, determined using a detergent compatible protein assay (DC Protein assay, BioRad). Finally, the sample was flash frozen in liquid nitrogen and stored at -80°C.

#### UapA-G411V

A modified version of UapA lacking the 11 N-terminal residues and containing a single point mutation UapA-G411V_Δ1–11_ was expressed as a fusion protein with the C-terminal tobacco etch virus (TEV) cleavage site followed by GFP and 8xHis tag. UapA-G411V_Δ1–11_ was transformed into the protease-deficient *Saccharomyces cerevisiae* strain FGY217 (MTAα, *ura3-52*, *lys2Δ201*, *pep4Δ*) described in detail elsewhere [80]. In brief, single colonies were inoculated in 10 mL minus-URA media [2 g/L amino acid mix w/o uracil, 6.7 g/L yeast nitrogen base w/o amino acids, 2% (w/v) glucose] in 50 mL aerated TubeSpin bioreactor tubes (TPP) and incubated at 30°C for 16 hours with 300 rpm shaking. The overnight cultures were diluted in 350 mL minus-URA media supplemented by 2% (w/v) glucose in a 1 L flask and incubated at 30°C for 24 hours, shaking at 300 rpm. The culture was then diluted with 1 L of minus-URA media supplemented with only 0.1% (w/v) glucose for induction at an OD_600_ of 0.6. 22 hours after induction with 2% (w/v) galactose, cells were harvested at 4,000 xg for 10 minutes and the pellets were resuspended in ~6 mL of CRB buffer [50 mM Tris (pH 7.5), 1 mM EDTA, and 0.6 M sorbitol] per litre of cell culture. The cells were lysed using a Constant systems cell disruptor at 25, 30, 33 and 36 kpsi (5°C). Cell debris was removed by centrifugation at 10,000 xg for 10 minutes at 4°C. The membranes were harvested by ultracentrifugation at 100,000 xg for 2 hours at 4°C and resuspended in 6 mL MRB buffer [20 mM Tris (pH 7.5), 0.3 M sucrose, 0.1 mM CaCl_2_] per litre of cell culture. Afterwards, membrane proteins were solubilised for 1 hour at 4°C in solubilisation buffer [1x PBS pH 7.5, 100 mM NaCl, 10% (v/v) glycerol, 1% (w/v) DDM, 1 mM xanthine, and one protease inhibitor tablet (Roche)] and insoluble matter was removed by centrifugation at 100,000 xg, for 45 minutes (4°C). The supernatant was supplemented with 10 mM imidazole (pH 7.5) and incubated for 2 hours at 4°C with 20 mL of nickel-nitrilotiacetic-acid (Ni-NTA) superflow resin (Quiagen) pre-washed in buffer A [1xPBS pH 7.5, 100 mM NaCl, 10 mM imidazole, 10% (v/v) glycerol, 0.03% (w/v) DDM, and 1 mM xanthine]. The mixture was loaded into an Econo-Pac gravity flow column (Bio-Rad) and washed with 6 CV of buffer A followed by 20 CV of Buffer A supplemented with 30 mM imidazole. The protein was eluted with 3 CV of buffer B [1x PBS pH 7.5, 150 mM NaCl, 10% glycerol, 310 mM imidazole, 1 mM xanthine, and 0.03% (w/v) DDM].

The protein concentration was estimated by measuring GFP fluorescence (Molecular Devices Spectramax M2) and TEV protease was added at a 1:1 ration of protease to protein. The sample was transferred to a dialysis membrane with a 12 kDa MWCO (Fisher Scientific) for 16 hours at 4°C for GFP-His tag cleavage. Following cleavage, the sample was supplemented with 12 mM imidazole, centrifuged for 10 minutes at 4,000 xg (4°C) and passed through a 0.22 μm filter to remove any precipitation. Afterwards, the sample was loaded on a 5 mL HisTrap column (GE Healthcare) pre-equilibrated with buffer C [20 mM Tris pH 7.5, 150 mM NaCl, 0.6 mM xanthine, and 0.03% (w/v) DDM] additionally containing 15 mM imidazole to remove TEV and cleavage product from the sample. The flow through was collected and concentrated to a volume of 0.5 mL in the Amicon 100 kDa MWCO concentrators (Merck) and injected into an equilibrated (buffer C) Superdex 200 10/300 gel filtration column (GE Healthcare). The sample was run at a flow rate of 0.35 mL/minute at 4°C. The quality of eluted fractions was checked by SDS-PAGE analysis (S1 Fig). The purest and most monodispersed fractions were pooled together, concentrated to 0.5 mg/mL using the Amicon 100 kDa MWCO concentrators (Merck) and flash frozen in liquid nitrogen for storage at -80°C until used.

#### *Tm*-PPase

Protein expression and purification followed protocols described in detail elsewhere [73]. In brief, 6xHis-tagged *Tm*-PPase in the pRS1024 vector under the control of the PMA1 promoter was freshly transformed into the protease-deficient *Saccharomyces cerevisiae* strain BJ1991 (α *prb1-1122 pep4-3 leu2 trp1 ura3-52 gal2*) following a standard protocol for yeast transformation [80]. Colonies grown on yeast-peptone-dextrose (YPD) plates [1% (w/v) yeast extract, 2% (w/v) peptone, 2% (w/v) dextrose, 1.5% (w/v) agar, 100 μg/mL Carbenicillin] were transferred into 250 mL of synthetic Complete Dropout minus leucine (SCD-Leu) media [2.7% (w/v) yeast nitrogen base, 2% (w/v) glucose, 0.5 mM L-tryptophan, 0.4 mM L-histidine, 0.2 mM L-adenine, 0.2 mM L-uracil, 100 μg/mL Carbenicillin]. The culture was cultivated for 24 hours (30°C, 200 rpm) and used to inoculate 750 mL 1.5X YPD media for protein expression (8 hours, 30°C, 200 rpm). Afterwards, cells were harvested and washed twice in deionised water. The cell pellets were resuspended in 0.5 mL buffer [200 mM Tris pH 7.5, 40% (w/v) glycerol, 10 mM EDTA, 2 mM dithiothreitol (DTT), 0.2 mM phenylmethylsulfonyl fluoride (PMSF)] per gram of dry cell mass for lysis using a bead beater and 0.5-mm glass beads. The bead beater chamber was topped up with 10 mM Tris pH 7.5, 10% (v/v) glycerol, 5 mM EDTA, and 1 mM DTT until completely full. Cells were lysed at 4°C by 12x 1-minute activations, interspaced by 1-minute cool-down periods on ice. Cell debris was removed (3,500 xg, 4°C, 15 minutes) and the supernatant was diluted with 10 mM Tris pH 7.5, 5 mM EDTA, and 1 mM DTT to a glycerol concentration of 20% (v/v). Membranes were harvested at 100,000 xg and 4°C for 1 hour and resuspended in 50 mM MES-NaOH pH 6.5, 20% (v/v) glycerol, 50 mM KCl, 5 mM MgCl_2_, 1.33 mM DTT, 0.336 mM PMSF, and 2 μg/mL Pepstatin A. The total protein concentration of the extracted membranes was determined in a Bradford assay and diluted to 7.2 mg/mL. Diluted membranes were supplemented with 0.33 mM Na_2_PP_i_ and mixed at a 3:1 ratio with 4x solubilisation buffer [50 mM MES-NaOH pH 6.5, 20% glycerol, and 5.34% (w/v) DDM]. Protein solubilisation followed the hot solve method [73] at 75°C for 1.5 hours. Denaturated proteins and insoluble matter were removed by centrifugation (4,000 xg, 15 minutes) and KCl was added to a final concentration of 0.3 M. 10 μL of Ni-NTA superflow resin was added per 1 mL of solubilised membrane sample. The mixture was loaded into an Econo-Pac gravity flow column after 1.5 hours at 40°C and 200 rpm shaking. The resin was washed with 2x CV of wash buffer [50 mM MES-NaOH pH 6.5, 20% (w/v) glycerol, 50 mM KCl, 5 mM MgCl_2_, 20 mM imidazole, 0.05% (w/v) octyl glucose neopentyl glycol (OGNG), 1 mM DTT, 0.2 mM PMSF, and 2 μg/μL pepstatin A] followed by protein elution in 2x CV of 50 mM MES-NaOH, pH 6.5, 3.5% (v/v) glycerol, 50 mM KCl, 5 mM MgCl_2_, 400 mM imidazole, 0.05% (w/v) OGNG, and 1 mM DTT. Imidazole was removed in a buffer exchange into 50 mM MES-NaOH, pH 6.5, 3.5% (v/v) glycerol, 50 mM KCl, 5 mM MgCl_2_, 0.05% (w/v) OGNG) using a PD10 desalting column. The sample purity was checked by SDS-PAGE analysis (S1 Fig). Purified protein was concentrated to 1 mg/mL in 50 kDa MWCO Vivaspin 2 centrifugal concentrators, flash frozen in liquid nitrogen and stored at -80°C until used.

### Relipidation of membrane proteins

The lipid screen was thawed and centrifuged for 3 minutes at 1,000 xg prior to use to ensure all lipid powder was at the bottom of the plate. 3x protein buffers without detergent (A_2A_R: 30 mM HEPES pH 7.5, 2400 mM NaCl, UapA: 60 mM Tris pH 7.5, 450 mM NaCl, 1.8 mM xanthine; *Tm*-PPase: 150 mM MES-NaOH, pH 6.5, 11.5% (v/v) glycerol, 150 mM KCl, 15 mM MgCl_2_) were prepared, degassed and cooled down to 4°C. To each well containing 0.3 mg lipids, 50 μL 3% (w/v) detergent (OGNG for *Tm*-PPase, DDM for UapA and A_2A_R) was added. Solubilisation of the lipids took place overnight at 24°C while shaking at 250 rpm. This was aided by pipetting up and down 10x using a multi-channel pipette. The RAMP lipid screen was centrifuged for 3 minutes at 1,000 xg to collect all undissolved lipids at the bottom of the plate. 15 μL of solubilised lipids (supernatant) was mixed with 15 μL of water and 15 μL of the respective 3x protein buffer, diluting the detergent and lipid concentration to 1% and 2 mg/mL, respectively. The remaining lipid screen was sealed and stored at -20°C in the dark for future use. Protein at 1 mg/mL (*Tm*-PPase, A_2A_R) or 0.5 mg/mL UapA was mixed 1:1 with the lipid supplemented 1x protein buffer. The required sample volume for stability testing using the Prometheus NT.48 nanoDSF (Nanotemper) was 8–10 μL per measurement.

### Differential scanning fluorimetry for stability testing (nanoDSF)

8–10 μL of the prepared protein samples were transferred into standard grade Prometheus NT.48 capillaries (Nanotemper). The capillaries were loaded into the Prometheus NT.48 and the excitation power of the device was adjusted to give a fluorescence signal at 330 nm just below 20000 RFU. The PR.ThermControl (version 2.1.2) software was used to set up a melting scan from 20°C to 99°C with a ramp rate of 1°C/min. The fluorescence emission at 330 nm (F_330_), 350 nm (F_350_), the 350:330 ratio, and the light scattering signal were recorded over the course of the melting scan. The effect of all lipids on protein stability was tested in the same run for each protein and compared to the reference sample without lipid added (apo). Additionally, another control with a known ligand added to the sample was included for each protein (*Tm*-PPase: imidodiphosphate (IDP), UapA: xanthine, A_2A_R: ZM241385). The experiment was repeated three times for each protein to calculate the standard error of the mean (SEM) stated in the text and figures. The apparent melting temperatures (T_m_) were obtained at the minimum/maximum of the first derivative of the F_330_ signal or 350:330 ratio by the PR.ThermControl software. The data was exported for plotting and analysis in GraphPad Prism 7.0.

## Supporting information

S1 Fig Purified membrane proteins used in the stability screening with added lipids from the lipid screen. A Coomassie-stained SDS-PAGE gel is shown for each tested protein in an individual panel. The black arrow indicates the band corresponding to the target protein. In the purified A_2A_R sample, more than one A_2A_R species is present due to glycosylation, which is commonly observed for this protein.
(DOCX)

S2 Fig A_2A_R purification gel image: Used to generate the first panel in S1 Fig (also shown on left). All lanes that were removed from this original image are indicated by an X.
(DOCX)

S3 Fig UapA purification gel image: Used to generate the second panel in S1 Fig (also shown on left). All lanes that were removed from this original image are indicated by an X.
(DOCX)

S4 Fig *Tm*-PPase purification gel image: Used to generate the third panel in S1 Fig (also shown on left). All lanes that were removed from this original image are indicated by an X.
(DOCX)

S1 Raw images A2AR purification gel image: Used to generate left panel in S1 Fig (also shown below). (PDF)

## References

[1] Uhlén M. et al. Tissue-based map of the human proteome. *Science* 347, (2015). doi: 10.1126/science.1260419 25613900

[2] Sriram K. & Insel P. A. G Protein-Coupled Receptors as Targets for Approved Drugs: How Many Targets and How Many Drugs? *Mol*. *Pharmacol*. 93, 251–258 (2018). doi: 10.1124/mol.117.111062 29298813 PMC5820538

[3] Yang D. et al. G protein-coupled receptors: structure- and function-based drug discovery. *Sig*. *Transduct*. *Target Ther*. 6, 1–27 (2021). doi: 10.1038/s41392-020-00435-w 33414387 PMC7790836

[4] Kwan T. O. C., Axford D. & Moraes I. Membrane protein crystallography in the era of modern structural biology. *Biochem*. *Soc*. *Trans*. 48, 2505–2524 (2020). doi: 10.1042/BST20200066 33170253

[5] Hardy D., Bill R. M., Jawhari A. & Rothnie A. J. Overcoming bottlenecks in the membrane protein structural biology pipeline. *Biochem*. *Soc*. *Trans*. 44, 838–844 (2016). doi: 10.1042/BST20160049 27284049

[6] Lundbæk J. A., Collingwood S. A., Ingólfsson H. I., Kapoor R. & Andersen O. S. Lipid bilayer regulation of membrane protein function: gramicidin channels as molecular force probes. *J*. *Royal Soc*. *Interface* 7, 373–395 (2010). doi: 10.1098/rsif.2009.0443 19940001 PMC2842803

[7] Laganowsky A. et al. Membrane proteins bind lipids selectively to modulate their structure and function. *Nature* 510, 172–175 (2014). doi: 10.1038/nature13419 24899312 PMC4087533

[8] Seddon A. M., Curnow P. & Booth P. J. Membrane proteins, lipids and detergents: not just a soap opera. *Biochim*. *Biophys*. *Acta Biomembr*. 1666, 105–117 (2004). 10.1016/j.bbamem.2004.04.011 15519311

[9] Huang H., Ge B., Sun C., Zhang S. & Huang F. Membrane curvature affects the stability and folding kinetics of bacteriorhodopsin. *Process Biochem*. 76, 111–117 (2019).

[10] Marsh D. Lateral Pressure Profile, Spontaneous Curvature Frustration, and the Incorporation and Conformation of Proteins in Membranes. *Biophys*. *J*. 93, 3884–3899 (2007). doi: 10.1529/biophysj.107.107938 17704167 PMC2084255

[11] Kapsalis C. et al. Allosteric activation of an ion channel triggered by modification of mechanosensitive nano-pockets. *Nat*. *Commun*. 10, 1–14 (2019). doi: 10.1038/s41467-018-07882-8 31601809 PMC6787021

[12] Pyle E. et al. Structural Lipids Enable the Formation of Functional Oligomers of the Eukaryotic Purine Symporter UapA. *Cell Chem*. *Biol*. 25, 840–848.e4 (2018). doi: 10.1016/j.chembiol.2018.03.011 29681524 PMC6058078

[13] Gupta K. et al. The role of interfacial lipids in stabilizing membrane protein oligomers. *Nature* 541, 421–424 (2017). doi: 10.1038/nature20820 28077870 PMC5501331

[14] Sejdiu B. I. & Tieleman D. P. Lipid-Protein Interactions Are a Unique Property and Defining Feature of G Protein-Coupled Receptors. *Biophys*. *J*. 118, 1887–1900 (2020). doi: 10.1016/j.bpj.2020.03.008 32272057 PMC7175695

[15] Song W., Yen H.-Y., Robinson C. V. & Sansom M. S. P. State-dependent Lipid Interactions with the A_2a_ Receptor Revealed by MD Simulations Using *In Vivo*-Mimetic Membranes. *Structure* 27, 392–403.e3 (2019). doi: 10.1016/j.str.2018.10.024 30581046 PMC7031699

[16] Scott D. J., Kummer L., Tremmel D. & Plückthun A. Stabilizing membrane proteins through protein engineering. *Curr*. *Opin*. *Chem*. *Biol*. 17, 427–435 (2013). doi: 10.1016/j.cbpa.2013.04.002 23639904

[17] Bhattacharya S., Lee S., Grisshammer R., Tate C. G. & Vaidehi N. Rapid Computational Prediction of Thermostabilizing Mutations for G Protein-Coupled Receptors. *J*. *Chem*. *Theory Comput*. 10, 5149–5160 (2014). doi: 10.1021/ct500616v 25400524 PMC4230369

[18] Popov P. et al. Computational design of thermostabilizing point mutations for G protein-coupled receptors. *eLife* 7, (2018). doi: 10.7554/eLife.34729 29927385 PMC6013254

[19] Harborne S. P. D. et al. IMPROvER: the Integral Membrane Protein Stability Selector. *Sci*. *Rep*. 10, 15165 (2020). doi: 10.1038/s41598-020-71744-x 32938971 PMC7495477

[20] Tribet C., Audebert R. & Popot J.-L. Amphipols: Polymers that keep membrane proteins soluble in aqueous solutions. *Proc*. *Natl*. *Acad*. *Sci*. *U*.*S*.*A*. 93, 15047–15050 (1996). doi: 10.1073/pnas.93.26.15047 8986761 PMC26353

[21] Sanders C. R. & Prosser R. S. Bicelles: a model membrane system for all seasons? *Structure* 6, 1227–1234 (1998). doi: 10.1016/s0969-2126(98)00123-3 9782059

[22] Rigaud J.-L., Pitard B. & Levy D. Reconstitution of membrane proteins into liposomes: application to energy-transducing membrane proteins. *Biochim*. *Biophys*. *Acta Bioenerg*. 1231, 223–246 (1995). doi: 10.1016/0005-2728(95)00091-v 7578213

[23] Denisov I. G. & Sligar S. G. Nanodiscs for structural and functional studies of membrane proteins. *Nat*. *Struct*. *Mol*. *Biol*. 23, 481–486 (2016). doi: 10.1038/nsmb.3195 27273631 PMC8934039

[24] Carlson M. L. et al. The Peptidisc, a simple method for stabilizing membrane proteins in detergent-free solution. *eLife* 7, e34085 (2018). doi: 10.7554/eLife.34085 30109849 PMC6093710

[25] Knowles T. J. et al. Membrane Proteins Solubilized Intact in Lipid Containing Nanoparticles Bounded by Styrene Maleic Acid Copolymer. *J*. *Am*. *Chem*. *Soc*. 131, 7484–7485 (2009). doi: 10.1021/ja810046q 19449872

[26] Lee S. C. et al. A method for detergent-free isolation of membrane proteins in their local lipid environment. *Nat*. *Protoc*. 11, 1149–1162 (2016). doi: 10.1038/nprot.2016.070 27254461

[27] Kurisu G., Zhang H., Smith J. L. & Cramer W. A. Structure of the Cytochrome b_6_f Complex of Oxygenic Photosynthesis: Tuning the Cavity. *Science* 302, 1009–1014 (2003). doi: 10.1126/science.1090165 14526088

[28] Toyoshima C., Nakasako M., Nomura H. & Ogawa H. Crystal structure of the calcium pump of sarcoplasmic reticulum at 2.6 Å resolution. *Nature* 405, 647–655 (2000). doi: 10.1038/35015017 10864315

[29] Long S. B., Campbell E. B. & MacKinnon R. Crystal Structure of a Mammalian Voltage-Dependent *Shaker* Family K^+^ Channel. *Science* 309, 897–903 (2005). doi: 10.1126/science.1116269 16002581

[30] Guan L., Smirnova I. N., Verner G., Nagamori S. & Kaback H. R. Manipulating phospholipids for crystallization of a membrane transport protein. *Proc*. *Natl*. *Acad*. *Sci*. *U*.*S*.*A*. 103, 1723–1726 (2006). doi: 10.1073/pnas.0510922103 16446422 PMC1413674

[31] Cherezov V. et al. High Resolution Crystal Structure of an Engineered Human β_2_-Adrenergic G protein-Coupled Receptor. *Science* 318, 1258–1265 (2007). doi: 10.1126/science.1150577 17962520 PMC2583103

[32] Shah N. R., Vidilaseris Keni., Xhaard H. & Goldman A. Integral membrane pyrophosphatases: a novel drug target for human pathogens? *AIMS Biophys*. 3, 171–194 (2016).

[33] Kousha M., Tadi R. & Soubani A. O. Pulmonary aspergillosis: a clinical review. *Eur*. *Respir*. *Rev*. 20, 156–174 (2011). doi: 10.1183/09059180.00001011 21881144 PMC9584108

[34] Lappas C. M., Sullivan G. W. & Linden J. Adenosine A_2A_ agonists in development for the treatment of inflammation. *Expert Opin*. *Investig*. *Drugs* 14, 797–806 (2005). doi: 10.1517/13543784.14.7.797 16022569

[35] Takahashi M., Fujita M., Asai N., Saki M. & Mori A. Safety and effectiveness of istradefylline in patients with Parkinson’s disease: interim analysis of a post-marketing surveillance study in Japan. *Expert Opin*. *Pharmacother* 19, 1635–1642 (2018). doi: 10.1080/14656566.2018.1518433 30281377

[36] Holmes A. O. M., Kalli A. C. & Goldman A. The Function of Membrane Integral Pyrophosphatases From Whole Organism to Single Molecule. *Front*. *Mol*. *Biosci*. 6, (2019). doi: 10.3389/fmolb.2019.00132 31824962 PMC6882861

[37] Park S. et al. Up-regulation of a H^+^-pyrophosphatase (H+-PPase) as a strategy to engineer drought-resistant crop plants. *Proc*. *Natl*. *Acad*. *Sci*. *U*.*S*.*A*. 102, 18830–18835 (2005). doi: 10.1073/pnas.0509512102 16361442 PMC1323196

[38] Lesk C., Rowhani P. & Ramankutty N. Influence of extreme weather disasters on global crop production. *Nature* 529, 84–87 (2016). doi: 10.1038/nature16467 26738594

[39] Gournas C., Oestreicher N., Amillis S., Diallinas G. & Scazzocchio C. Completing the purine utilisation pathway of *Aspergillus nidulans*. *Fungal Genet*. *Biol*. 48, 840–848 (2011). doi: 10.1016/j.fgb.2011.03.004 21419234

[40] Gournas C., Papageorgiou I. & Diallinas G. The nucleobase–ascorbate transporter (NAT) family: genomics, evolution, structure–function relationships and physiological role. *Mol. BioSyst*. 4, 404–416 (2008). doi: 10.1039/b719777b 18414738

[41] Jacobson K. A. Introduction to Adenosine Receptors as Therapeutic Targets. *Handb*. *Exp*. *Pharmacol*. 1–24 (2009). doi: 10.1007/978-3-540-89615-9_1 19639277 PMC3415694

[42] Gourdon P. et al. HiLiDe—Systematic Approach to Membrane Protein Crystallization in Lipid and Detergent. *Crystal Growth & Design* 11, 2098–2106 (2011).

[43] Hénault C. M. et al. A lipid site shapes the agonist response of a pentameric ligand-gated ion channel. *Nat*. *Chem*. *Biol*. 15, 1156–1164 (2019). doi: 10.1038/s41589-019-0369-4 31591563 PMC8423587

[44] McGoldrick L. L. et al. Structure of the thermo-sensitive TRP channel TRP1 from the alga *Chlamydomonas reinhardtii*. *Nat. Commun*. 10, 4180 (2019). doi: 10.1038/s41467-019-12121-9 31519888 PMC6744473

[45] Vitrac H., Mallampalli V. K. P. S., Bogdanov M. & Dowhan W. The lipid-dependent structure and function of LacY can be recapitulated and analyzed in phospholipid-containing detergent micelles. *Sci. Rep*. 9, 11338 (2019). doi: 10.1038/s41598-019-47824-y 31383935 PMC6683142

[46] Broecker J., Eger B. T. & Ernst O. P. Crystallogenesis of Membrane Proteins Mediated by Polymer-Bounded Lipid Nanodiscs. *Structure* 25, 384–392 (2017). doi: 10.1016/j.str.2016.12.004 28089451

[47] Srivastava S. R., Zadafiya P. & Mahalakshmi R. Hydrophobic Mismatch Modulates Stability and Plasticity of Human Mitochondrial VDAC2. *Biophys*. *J*. 115, 2386–2394 (2018). doi: 10.1016/j.bpj.2018.11.001 30503532 PMC6301912

[48] Baturin S., Galka J. J., Piyadasa H., Gajjeraman S. & O’Neil J. D. The effects of a protein osmolyte on the stability of the integral membrane protein glycerol facilitator. *Biochem*. *Cell Biol*. 92, 564–575 (2014). doi: 10.1139/bcb-2014-0076 25387032

[49] Koshy C. et al. Structural evidence for functional lipid interactions in the betaine transporter BetP. *EMBO J*. 32, 3096–3105 (2013). doi: 10.1038/emboj.2013.226 24141878 PMC3844952

[50] Pyle E. et al. Protein–Lipid Interactions Stabilize the Oligomeric State of BOR1p from *Saccharomyces cerevisiae*. *Anal*. *Chem*. 91, 13071–13079 (2019). doi: 10.1021/acs.analchem.9b03271 31513392

[51] Schrecke S. et al. Selective regulation of human TRAAK channels by biologically active phospholipids. *Nat*. *Chem*. *Biol*. 17, 89–95 (2021). doi: 10.1038/s41589-020-00659-5 32989299 PMC7746637

[52] Dalen A. van, Hegger, S., Killian, J. A. & Kruijff, B. de. Influence of lipids on membrane assembly and stability of the potassium channel KcsA. *FEBS Lett*. 525, 33–38 (2002). doi: 10.1016/s0014-5793(02)03061-2 12163157

[53] Jaakola V.-P. et al. The 2.6 Angstrom Crystal Structure of a Human A_2A_ Adenosine Receptor Bound to an Antagonist. *Science* 322, 1211–1217 (2008). doi: 10.1126/science.1164772 18832607 PMC2586971

[54] Laursen L. et al. Cholesterol binding to a conserved site modulates the conformation, pharmacology, and transport kinetics of the human serotonin transporter. *J*. *Biol*. *Chem*. 293, 3510–3523 (2018). doi: 10.1074/jbc.M117.809046 29352106 PMC5846164

[55] Nji E., Chatzikyriakidou Y., Landreh M. & Drew D. An engineered thermal-shift screen reveals specific lipid preferences of eukaryotic and prokaryotic membrane proteins. *Nat*. *Commun*. 9, 1–12 (2018). doi: 10.1038/s41467-017-02088-w 30315156 PMC6185904

[56] Hattori M., Hibbs R. E. & Gouaux E. A fluorescence-detection size-exclusion chromatography-based thermostability assay to identify membrane protein expression and crystallization conditions. *Structure* 20, 1293–1299 (2012). doi: 10.1016/j.str.2012.06.009 22884106 PMC3441139

[57] Misquitta L. V. et al. Membrane protein crystallization in lipidic mesophases with tailored bilayers. *Structure* 12, 2113–2124 (2004). doi: 10.1016/j.str.2004.09.020 15576026

[58] Landau E. M. & Rosenbusch J. P. Lipidic cubic phases: A novel concept for the crystallization of membrane proteins. *Proc*. *Natl*. *Acad*. *Sci*. *U*.*S*.*A*. 93, 14532–14535 (1996). doi: 10.1073/pnas.93.25.14532 8962086 PMC26167

[59] Deng Z. et al. Cryo-EM and X-ray structures of TRPV4 reveal insight into ion permeation and gating mechanisms. *Nat*. *Struct*. *Mol*. *Biol*. 25, 252–260 (2018). doi: 10.1038/s41594-018-0037-5 29483651 PMC6252174

[60] Zubcevic L. et al. Cryo-electron microscopy structure of the TRPV2 ion channel. *Nat*. *Struct*. *Mol*. *Biol*. 23, 180–186 (2016). doi: 10.1038/nsmb.3159 26779611 PMC4876856

[61] Jin P. et al. Electron cryo-microscopy structure of the mechanotransduction channel NOMPC. *Nature* 547, 118–122 (2017). doi: 10.1038/nature22981 28658211 PMC5669069

[62] Sharom F. J. Complex Interplay between the P-Glycoprotein Multidrug Efflux Pump and the Membrane: Its Role in Modulating Protein Function. *Front*. *Oncol*. 4, (2014). 24624364 10.3389/fonc.2014.00041 PMC3939933

[63] Jungnickel K. E. J., Parker J. L. & Newstead S. Structural basis for amino acid transport by the CAT family of SLC7 transporters. *Nat*. *Commun*. 9, 550 (2018). doi: 10.1038/s41467-018-03066-6 29416041 PMC5803215

[64] Palsdottir H. & Hunte C. Lipids in membrane protein structures. *Biochim*. *Biophys*. *Acta Biomembr*. 1666, 2–18 (2004). doi: 10.1016/j.bbamem.2004.06.012 15519305

[65] Zhang Y. et al. Visualization of the mechanosensitive ion channel MscS under membrane tension. *Nature* 590, 509–514 (2021). doi: 10.1038/s41586-021-03196-w 33568813

[66] Hanson M. A. et al. A Specific Cholesterol Binding Site Is Established by the 2.8 Å Structure of the Human β_2_-Adrenergic Receptor. *Structure* 16, 897–905 (2008). doi: 10.1016/j.str.2008.05.001 18547522 PMC2601552

[67] Gimpl G. & Fahrenholz F. Cholesterol as stabilizer of the oxytocin receptor. *Biochim*. *Biophys*. *Acta Biomembr*. 1564, 384–392 (2002). doi: 10.1016/s0005-2736(02)00475-3 12175921

[68] Oates J. et al. The role of cholesterol on the activity and stability of neurotensin receptor 1. *Biochim*. *Biophys*. *Acta Biomembr*. 1818, 2228–2233 (2012). doi: 10.1016/j.bbamem.2012.04.010 22551944

[69] Liu W. et al. Structural Basis for Allosteric Regulation of GPCRs by Sodium Ions. *Science* 337, 232–236 (2012). doi: 10.1126/science.1219218 22798613 PMC3399762

[70] Parks L. W. & Casey W. M. Physiological implications of sterol biosynthesis in yeast. *Annu*. *Rev*. *Microbiol*. 49, 95–116 (1995). doi: 10.1146/annurev.mi.49.100195.000523 8561481

[71] Cournia Z., Ullmann G. M. & Smith J. C. Differential Effects of Cholesterol, Ergosterol and Lanosterol on a Dipalmitoyl Phosphatidylcholine Membrane: A Molecular Dynamics Simulation Study. *J*. *Phys*. *Chem*. *B* 111, 1786–1801 (2007). doi: 10.1021/jp065172i 17261058

[72] Alguel Y. et al. Structure of eukaryotic purine/H^+^ symporter UapA suggests a role for homodimerization in transport activity. *Nat*. *Commun*. 7, 11336 (2016). doi: 10.1038/ncomms11336 27088252 PMC4837479

[73] López-Marqués R. L. et al. Large-scale purification of the proton pumping pyrophosphatase from Thermotoga maritima: A ‘Hot-Solve’ method for isolation of recombinant thermophilic membrane proteins. *Biochim*. *Biophys*. *Acta Biomembr*. 1716, 69–76 (2005). doi: 10.1016/j.bbamem.2005.08.004 16182234

[74] Vidilaseris K. et al. Asymmetry in catalysis by Thermotoga maritima membrane-bound pyrophosphatase demonstrated by a nonphosphorus allosteric inhibitor. *Sci*. *Adv*. 5, eaav7574 (2019). doi: 10.1126/sciadv.aav7574 31131322 PMC6530997

[75] Heerklotz H. Interactions of surfactants with lipid membranes. *Q*. *Rev*. *Biophys*. 41, 205–264 (2008). doi: 10.1017/S0033583508004721 19079805

[76] Kulig W. et al. Experimental determination and computational interpretation of biophysical properties of lipid bilayers enriched by cholesteryl hemisuccinate. *Biochim*. *Biophys*. *Acta Biomembr*. 1848, 422–432 (2015). doi: 10.1016/j.bbamem.2014.10.032 25450348

[77] Kellosalo J., Kajander T., Kogan K., Pokharel K. & Goldman A. The Structure and Catalytic Cycle of a Sodium-Pumping Pyrophosphatase. *Science* 337, 473–476 (2012). doi: 10.1126/science.1222505 22837527

[78] Alexandrov A. I., Mileni M., Chien E. Y. T., Hanson M. A. & Stevens R. C. Microscale fluorescent thermal stability assay for membrane proteins. *Structure* 16, 351–359 (2008). doi: 10.1016/j.str.2008.02.004 18334210

[79] Weiß H. M. & Grisshammer R. Purification and characterization of the human adenosine A_2a_ receptor functionally expressed in *Escherichia coli*. *Eur*. *J*. *Biochem*. 269, 82–92 (2002). doi: 10.1046/j.0014-2956.2002.02618.x 11784301

[80] Gietz R. D. & Schiest R. H. High-efficiency yeast transformation using the LiAc/SS carrier DNA/PEG method. *Nat*. *Protoc*. 2, 31–34 (2007). doi: 10.1038/nprot.2007.13 17401334

