# Supplementary material for: A novel high-throughput screen for identifying lipids that stabilise membrane proteins in detergent based solution
Source: PLoS One. 2021 Jul 12;16(7):e0254118. doi: 10.1371/journal.pone.0254118 (PMC8274869; doi:10.1371/journal.pone.0254118)
Supplement: S1 Fig — A Coomassie-stained SDS-PAGE gel is shown for each tested protein in an individual panel. The black arrow indicates the band corresponding to the target protein. In the purified A2AR sample, more than one A2AR species is present due to glycosylation, which is commonly observed for this protein. (DOCX) [file pone.0254118.s001.docx]

# Cecchetti et al. Supporting information


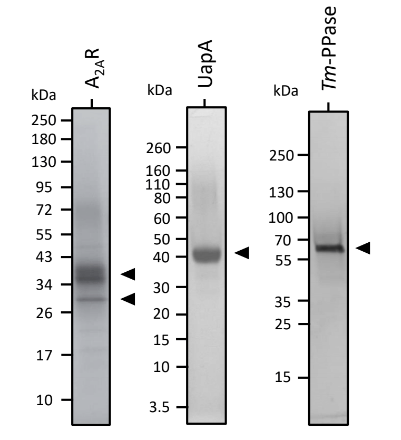


Figure S1: Purified membrane proteins used in the stability screening with added lipids from the lipid screen. A Coomassie-stained SDS-PAGE gel is shown for each tested protein in an individual panel. The black arrow indicates the band corresponding to the target protein. In the purified A_2A_R sample, more than one A_2A_R species is present due to glycosylation, which is commonly observed for this protein.
