# Supplementary material for: A novel high-throughput screen for identifying lipids that stabilise membrane proteins in detergent based solution
Source: PLoS One. 2021 Jul 12;16(7):e0254118. doi: 10.1371/journal.pone.0254118 (PMC8274869; doi:10.1371/journal.pone.0254118)
Supplement: S4 Fig — All lanes that were removed from this original image are indicated by an X. (DOCX) [file pone.0254118.s004.docx]

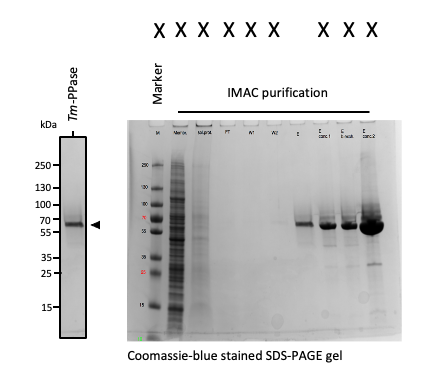


Figure S4 *Tm*-PPase purification gel image: used to generate the third panel in Figure S1 (also shown on left). All lanes that were removed from this original image are indicated by an X.
