# Supplementary material for: A novel high-throughput screen for identifying lipids that stabilise membrane proteins in detergent based solution
Source: PLoS One. 2021 Jul 12;16(7):e0254118. doi: 10.1371/journal.pone.0254118 (PMC8274869; doi:10.1371/journal.pone.0254118)
Supplement: S1 Raw images — (PDF) [file pone.0254118.s005.pdf]

$A_{2A}R$ purification gel image: used to generate left panel in Figure S1 (also shown below).

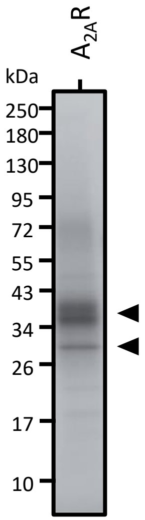

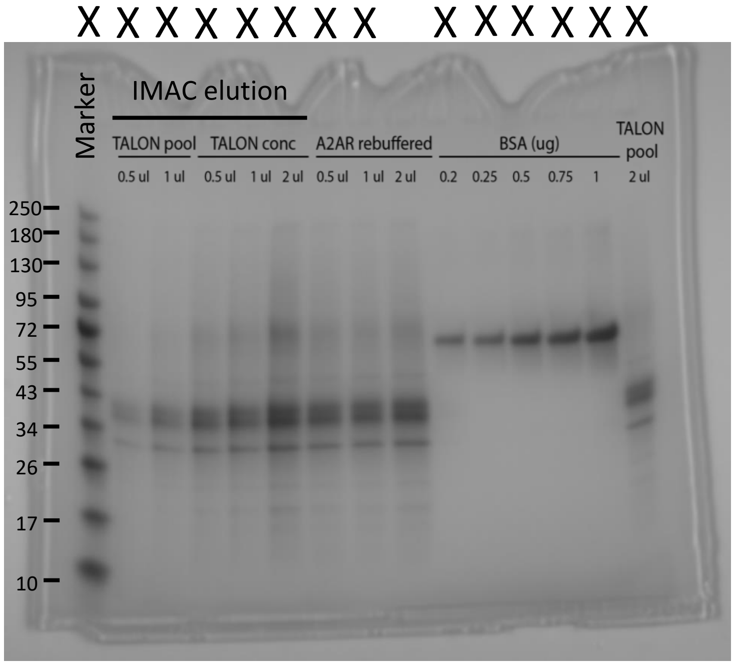

Coomassie-blue stained SDS-PAGE gel

UapA purification gel image: used to generate middel panel in Figure S1 (also shown below).

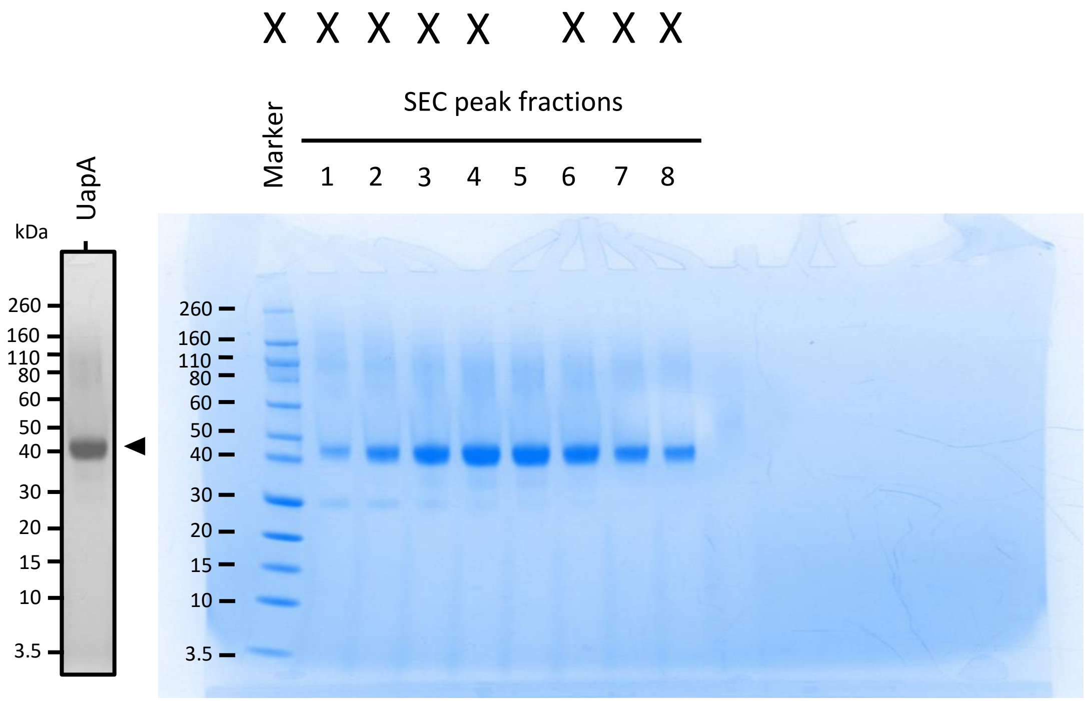

Coomassie-blue stained SDS-PAGE gel

Tm-PPase purification gel image: used to generate right panel in Figure S1 (also shown below).

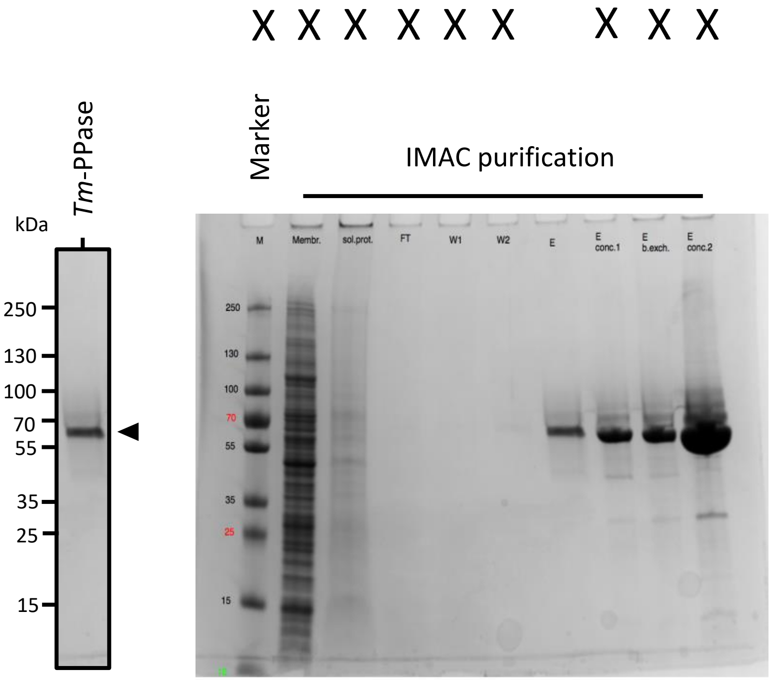

Coomassie-blue stained SDS-PAGE gel
